# Are Preoperative CT Findings Useful for Predicting Postoperative Intraabdominal Abscess in the Patients with Acute Appendicitis?

**DOI:** 10.3390/medicina55010006

**Published:** 2019-01-04

**Authors:** Atsushi Kohga, Kiyoshige Yajima, Takuya Okumura, Kimihiro Yamashita, Jun Isogaki, Kenji Suzuki, Katsuaki Muramatsu, Akira Komiyama, Akihiro Kawabe

**Affiliations:** 1 Division of Surgery, Fujinomiya City General Hospital, Nishiki-cho, Fujinomiya, Shizuoka 4180076, Japan; yaji@aurora.ocn.ne.jp (K.Y.); takuya330jp@yahoo.co.jp (T.O.); ymst13@yahoo.co.jp (K.Y.); jun_isogaki@hospital.fujinomiya.shizuoka.jp (J.I.); suzukik@hospital.fujinomiya.shizuoka.jp (K.S.); kaimai@hospital.fujinomiya.shizuoka.jp (A.K.); 2 Division of Radiology, Fujinomiya City General Hospital, Nishiki-cho, Fujinomiya, Shizuoka 4180076, Japan; nredmoon@gmail.com; 3 Division of Pathology, Fujinomiya City General Hospital, Nishiki-cho, Fujinomiya, Shizuoka 4180076, Japan; akomiyama-path@umin.ac.jp

**Keywords:** acute appendicitis, complicated appendicitis, laparoscopic appendectomy, intraabdominal abscess

## Abstract

*Background and objective*: In patients with acute appendicitis (AA), preoperative computed tomography (CT) findings suggesting development of intraabdominal abscess (IAA) had not been widely used. The aim of this study was to investigate the preoperative clinical and radiological factors that predict the development of a postoperative IAA in patients with AA who were treated by laparoscopic appendectomy (LA). *Methods*: Two hundred and sixteen patients with pathologically proven AA underwent LA between January 2013 and March 2018 in our department. Medical records and preoperative CT images of these 216 patients were retrospectively reviewed and the predictive factors of postoperative IAA were investigated. In addition, patients were divided into complicated appendicitis (CA) and simple appendicitis (SA) and perioperative factors of two groups were compared. *Results*: One hundred and forty-seven patients were diagnosed with CA, while the other 69 patients were diagnosed with SA. Sixteen patients developed postoperative IAA in the CA group, while no patients in the SA group did. The univariate analysis revealed that time from onset to surgery more than 3 days (*p* = 0.011), the preoperative CT finding of periappendiceal fluid (*p* = 0.003), abscess (*p* < 0.001), and free air (*p* < 0.001), operation time more than 120 min (*p* = 0.023) and placement of a drainage tube (*p* < 0.001) were significantly associated with the development of IAA. Multivariate analysis revealed that the preoperative CT finding of free air was independently associated with the development of IAA (*p* = 0.007, odds ratio = 5.427, 95% CI: 1.586–18.57). *Conclusions*: IAA developed predominantly in patients with CA. Preoperative CT findings of free air was found to be an independent predictor for the development of IAA. Surgeons should be meticulous in managing the postoperative course of patients with this finding.

## 1. Introduction

Recent meta-analyses have revealed the feasibility of laparoscopic appendectomy (LA) for acute appendicitis, even in those with complicated appendicitis (CA) [1,2].

Postoperative intraabdominal abscess (IAA) is one of the most severe complications after appendectomy. Some patients who develop IAA require a second surgery and/or percutaneous drainage, while some are treated conservatively [3,4,5]. Therefore, the development of postoperative IAA is a major cause of morbidity for patients who underwent appendectomy. To make a well-considered perioperative strategy associated with preventing or detecting the postoperative development of IAA, the perioperative prediction of risk factors for developing IAA is essential for surgeons.

Some risk factors associated with IAA have been reported [6,7,8]. However, there are few reports that report preoperative computed tomography (CT) findings related to the development of IAA. The aim of this study is to determine the predictive features of patients with a high risk of developing IAA after LA, identified based on their preoperative CT images.

## 2. Materials and Methods

### 2.1. Study Population

Two hundred and sixty-four patients, preoperatively diagnosed with acute appendicitis, underwent LA in our department between January 2013 and March 2018. The preoperative clinical and radiographic factors in these patients were retrospectively reviewed and patients were divided into simple a appendicitis (SA) group and a complicated appendicitis (CA) group. Patients who underwent interval appendectomy, patients who had been pathologically proven as having appendiceal tumor and patients who had not had preoperative CT were all excluded from this retrospective study. Perioperative factors in these groups were compared and the risk factors predicting postoperative IAA were investigated. The study was conducted in accordance with the Declaration of Helsinki, and the study protocol was approved by the institutional review board of Fujinomiya city general hospital (decision no. 83: 29 October 2018) and informed consent was waived for this retrospective study.

### 2.2. Definition of Simple and Complicated Appendicitis

Patients with findings of abscess, free air, or small bowel obstruction in the preoperative CT image were classified as having complicated appendicitis. In addition, patients with pathologically proven gangrenous appendicitis or perforated appendicitis were classified as having CA. All others were classified as having simple appendicitis. In the present study, 131 of the 216 patients were pathologically diagnosed with gangrenous appendicitis.

### 2.3. Surgical Strategy and Surgical Procedure

At our institution, all patients with an acute abdomen that suggested acute appendicitis underwent enhanced CT, unless the patient had a contraindication, such as an allergy to contrast agents or impaired renal function. Patients diagnosed with acute appendicitis were treated surgically or conservatively, according to patient’s tolerance and selection. For patients with generalized peritonitis, we performed an emergency operation. We generally intended to complete the operations laparoscopically for all patients preoperatively diagnosed with acute appendicitis after 2013.

We used a 10-mm port for the camera (30-degree oblique laparoscope) at the umbilicus incision and two 5-mm ports at the left and middle lower quadrants for instrumentation. For the patients in whom it was difficult to keep surgical field, another 5-mm port was added at the right lower quadrant. After pneumoperitoneum, dissection around the appendix was performed. The mesoappendix was cut using an ultrasonic dissector (Sonosurg; OLYMPUS, Tokyo, Japan). The root of the appendix was closed doubly by ligation, and the appendix was cut and removed. For patients in whom it was difficult to ligate the stump of the appendix, mobilization of the ileocecal was performed, and mini-laparotomy was added at the umbilicus incision to close the stump. In all cases with CA, intraabdominal irrigation with saline was performed after closing the stump until the contaminated fluids became clear. The drainage tube was placed on the right paracolic gutter and/or rectovesical pouch at the surgeon’s discretion. In seven cases in the SA group and four cases in the CA group, a single incisional laparoscopic appendectomy was performed at the surgeon’s discretion [9]. Generally, antibiotics were administered after surgery until fever and inflammatory response had subsided. Cefmetazole (2–4 g/d) or meropenem (1.5 g/d) were generally administered during the perioperative period. The selection of antimicrobial agents was made at the discretion of surgeons, taking the patient’s conditions into consideration.

Development of postoperative IAA was confirmed by CT image, which was performed selectively in patients with persistent or recurrent fever and/or inflammatory response after surgery.

### 2.4. Pre- and Perioperative Findings

Preoperative information included sex, age, history of appendicitis, American Society of Anesthesiologists Physical Status (ASA-PS) classification, WBC level, C-reactive protein (CRP) level, radiographic findings of preoperative CT imaging (appendicolith, fluid around appendix, abscess, free air, small bowel obstruction), time from onset to admission (days), time from CT to surgery (hours), and time from onset to surgery (days). The perioperative findings included operation time, postoperative length of hospital stay, conversion to laparotomy, placement of drainage tube, and incidence rates of postoperative complications and readmission. Complications were classified according to the grading system proposed by Dindo et al. [10].

### 2.5. Imaging Diagnostic Modality and Radiographic Parameters

**Figure 1:**
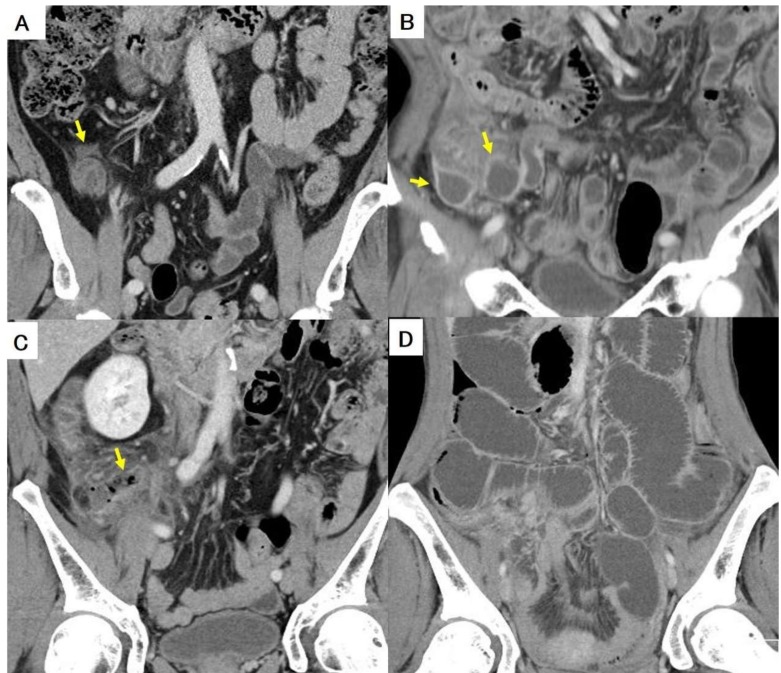
(**A**–**D**): Each figure shows presence of periappendiceal fluid (**A**), abscess (**B**), free air (**C**), and paralytic ileus (**D**). Arrows indicate each finding (**A**–**C**).

Among the 216 patients included in the study, all patients were preoperatively examined by abdominal MDCT in non-enhanced and/or enhanced phases using Light Speed VCT (GE Healthcare, Tokyo, Japan) before January 2017 and SOMATOM Force (Siemens Healthineers AG, Erlangen, Germany) from February 2017. Two experienced reviewers (a gastroenterological surgeon with twelve years of experience and a radiologist with seventeen years of experience) retrospectively reviewed the radiographic parameters to determine the presence of appendicolith, periappendiceal fluid, abscess, free air, and small bowel obstruction (Figure 1A–D). Measurements were performed in consensus and both readers were blinded to the outcome at the time of performing the measurements.

### 2.6. Statistical Analysis of the Surgical Outcomes and Clinical and Radiological Factors

The clinical and radiographic factors were compared. Each cutoff value was determined according to the median value or a receiver-operating characteristic curve, adjusting to a value easy to use in practice. The continuous variables’ distributions were verified using the Kolmogorov–Smirnov test and continuous variables were expressed as median and interquartile range because of their abnormal distributions. Pearson’s chi-square test was used to assess nominal variables, and continuous data were compared using the Mann–Whitney U test. Multivariate logistic regression analysis was performed to determine independent predictors of outcomes. All statistical analyses were performed using the Software Package for Social Sciences, version 11.5J for Windows 10 software program (SPSS, Chicago, IL, USA). A *p* value of <0.05 was considered significant.

## 3. Results

### 3.1. Comparison of the Clinical Factors between the Patients in the SA Group and CA Group 

**Figure 2:**
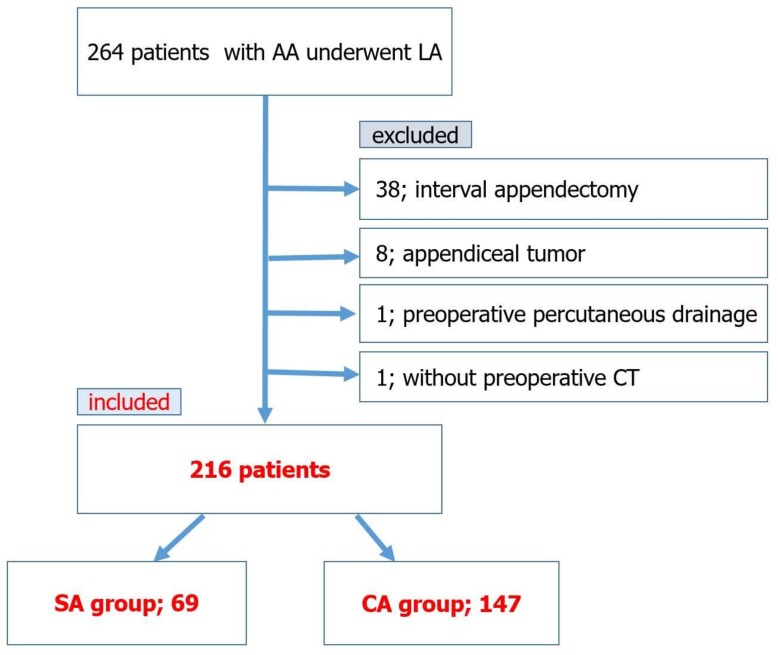
Conceptual model of the study. Finally, 216 patients met the study criteria and were included; 69 patients were classified as having SA (SA group; *n* = 69) and 147 patients were classified as having CA (CA group; *n* = 147).

In 264 patients, 38 patients underwent interval appendectomy, eight patients were pathologically diagnosed with appendiceal tumor (two were appendiceal cancer, six were mucinous neoplasm), one patient had a preoperatively percutaneous drainage performed, and one patient did not have a preoperative CT. These 48 patients were all excluded from this retrospective study and the other 216 patients met the study criteria and were included; 69 patients were classified as having SA (SA group; *n* = 69) and 147 patients were classified as having CA (CA group; *n* = 147) (Figure 2). The total of 216 patients included 135 males and 81 females. The mean age was 37.1 ± 22.9 (range 5 to 91). Clinical characteristics of the SA and CA groups are shown in Table 1. The mean age of the CA group was significantly higher than that of the SA group (*p* = 0.008). The presence of a history of appendicitis was significantly lower in the CA group (*p* < 0.001). The number of the patients with an ASA-PS classification of 2 or 3 was significantly higher in the CA group (*p* < 0.001). There were no significant differences in the preoperative WBC, while the mean CRP level was significantly higher in the CA group. Regarding preoperative CT findings, appendicolith, periappendiceal fluid, abscess, and free air were found significantly more often in the CA group (*p* < 0.001 respectively). The CT finding of small bowel obstruction was also found significantly more often in the CA group (*p* < 0.003). The time from operation to surgery was significantly longer in the CA group (*p* < 0.001). 

Perioperative outcomes are shown in Table 2. The operation time was significantly longer in the CA group (*p* = 0.005) and the postoperative length of stay was significantly longer in the CA group (*p* < 0.001). The incidence rate of conversion to laparotomy was not statistically significant. The number of patients who had drainage tubes placed was significantly higher in the CA group (*p* < 0.001). Regarding postoperative complications, the incidence rate of Grade 2 or more and Grade 3 were significantly higher in the CA group (*p* = 0.002, *p* = 0.009, respectively). Intraabdominal abscess developed in 16 patients (10.8%) of the CA group while no patients developed it in the SA group (*p* = 0.004). Three of these 16 patients required a second operation and nine patients required percutaneous drainage, while the other four patients were treated conservatively. The incident rate of superficial infection and paralytic ileus was not significantly different. Readmission associated with the first operation was required for 11 patients in the CA group (*p* = 0.019) within one year after discharge. The reason for readmission included IAA (*n* = 7), superficial infection (*n* = 1), strangulated ileus (*n* = 1), ventral hernia (*n* = 1), and abdominal pain (*n* = 1).

### 3.2. Perioperative Prediction of IAA in All Patients

Univariate analysis revealed that time from onset to surgery more than 3 days (*p* = 0.011), the preoperative CT finding of periappendiceal fluid (*p* = 0.003), abscess (*p* < 0.001), and free air (*p* < 0.001) were significantly associated with the development of IAA. In addition, operation time more than 120 min (*p* = 0.023) and placement of a drainage tube (*p* < 0.001) were significantly associated with the development of IAA (Table 3). Multivariate analysis revealed that the preoperative CT finding of free air was independently associated with the development of IAA (*p* = 0.007, odds ratio = 5.427, 95% CI: 1.586~18.57). Presence of free air in preoperative CT showed a 50.0% sensitivity, 93.0% specificity and 89.8% accuracy for the development of postoperative IAA.

## 4. Discussion

Our study revealed that IAA predominantly developed in patients with CA. Preoperative CT findings of free air was found to be an independent predictor for the development of IAA.

Previously, LA has been reported to imply a higher risk for developing IAA compared to that of conventional open appendectomy [11,12]. On the other hand, recent reports suggest that the risk for IAA is comparable between LA and open appendectomy [1,2,13,14,15,16]. These reports suggest that LA is already a treatment of choice for acute appendicitis, including CA. According to a recent meta-analysis, the incident rate of IAA in patients with CA after LA was 8.0% [1]. In the present study, 18 out of 147 patients with CA (12.2%) developed complications of Grade 3 and, notably, 12 of these 18 (66.6%) were derived from IAA. In addition, three patients required a second operation due to IAA. These results suggest that IAA is a major complication after LA.

Several risk factors for developing IAA, such as obesity, leukocytosis, perforated appendicitis, longer operation time, and peritoneal irrigation have been suggested previously [6,7,8]. On the other hand, preoperative CT findings that predict the postoperative development of IAA have not been investigated in detail. Kim et al. investigated the association between preoperative CT findings and 30-day adverse events. In their study, a presence of extraluminal air was not significantly associated with 30-day adverse events [17]. However, in their study, presence of extraluminal air showed a tendency to be found in patients with postoperative adverse event (*p* = 0.059 in univariate analysis). In addition, as opposed to our study, they included not only IAA but also peritonitis and small bowel obstruction as adverse event and their study population was limited to the patients with appendiceal inflammatory mass.

Our study also revealed that postoperative IAA predominantly develops in patients with CA. Although preoperative CT finding is considered useful for distinguish CA from SA [18,19,20,21], the definition of CA includes pathological findings, which is proven postoperatively. We retrospectively reviewed the preoperative CT findings of all study patients with acute appendicitis and found that the presence of free air was independently associated with the development of IAA, while the presence of abscess or periappendiceal fluid was not an independent predictor. We suppose that free air reflects a presence of major perforation in the appendix wall, resulting in bacterial adhesion which is not cleared by irrigation. We propose that this bacterial adhesion should be considered a contributing factor in abscess formation.

In our study, the placement of a drainage tube was also significantly associated with the development of IAA in patients with CA. However, in our study, we placed a drainage tube at the surgeon’s discretion; that is, we placed them in patients who were considered high risk for developing IAA according to intraoperative findings. Therefore, this result might imply a strong bias. It remains controversial whether a drainage tube is useful for preventing IAA. Some authors advocate that a drainage tube should be placed after peritoneal irrigation in order to prevent IAA [6]. On the other hand, according to a recent report, there is no clinical improvement by using an abdominal drainage after LA for CA [22,23]. An additional validation study is required regarding this aspect. 

Regarding intraoperative irrigation, Cho et al. reported that irrigation was a risk factor for developing IAA [6], and further that irrigation implies a risk for spreading contamination. However, it was not demonstrated in a recent meta-analysis [24]. In our study, all of the patients in the CA group performed irrigation until the contaminated ascites became clear. Therefore, we could not validate whether irrigation could be a risk factor for IAA.

Our study was not without limitations. First, this study is based on a retrospective and single-center experience. Another limitation was that the number of the patients who developed IAA was small and postoperative CT was selectively performed. Additional external validation is necessary to confirm that these findings are applicable to other patient groups. In addition, in our study, the patients were not distinguished according to their age, ranging 5 to 91 years. Inconsistency of antimicrobial agents might be another limitation. Furthermore, two readers obtained CT findings via a consensus read. It would be desirable to perform measurement by several readers independently.

## 5. Conslusions

We found that the presence of free air on preoperative CT in the patients with acute appendicitis was an independent predictor for postoperative development of IAA. Surgeons should be meticulous in managing the postoperative course of patients with this finding.

## Figures and Tables

**Table 1:** The results of the univariate analyses of preoperative clinical factors between SA and CA in the patients who underwent LA.

	SA Group (*n* = 69)	CA Group (*n* = 147)	Univariate Analysis *p*
Gender, *n* (male/female)	42/27	93/54	0.734
Age, median (range), years	27 (8–91)	40 (5–91)	0.008
History of appendicitis, *n* (%)	16 (23.1)	8 (5.4%)	<0.001
ASA-PS score; class 1/class 2 or 3	58/11	91/56	0.001
Preoperative WBC, median (range), 10^9^/L	13.8 (4.6–23.3)	14 (2.1–10.2)	0.139
Preoperative CRP, median (range), mg/dL	0.7 (0.0–21.4)	5.5 (0.0–51.9)	<0.001
Preoperative CT findings			
Appendicolith, *n* (%)	16 (23.1)	77 (52.3)	<0.001
Periappendiceal fluid *n* (%)	19 (27.5)	80 (54.4)	<0.001
Abscess *n* (%)	0	27 (18.3)	<0.001
Free air *n* (%)	0	22 (14.9)	<0.001
Small bowel obstruction *n* (%)	0	17 (11.4)	0.003
Time from onset to surgery, median (range), days	1 (0–7)	2 (0–27)	<0.001

SA, simple appendicitis; CA, complicated appendicitis; LA, laparoscopic appendectomy; ASA-PS, American Society of Anesthesiologists physical status. Pearson’s chi-square test was used to assess nominal variables, and continuous data were compared using the Mann–Whitney U test.

**Table 2:** The results of the univariate analyses of perioperative outcomes between SA and CA in the patients who underwent LA.

	SA Group (*n* = 69)	CA Group (*n* = 147)	Univariate Analysis *p*
Operation time, median (range), min	67 (33–158)	77 (34–252)	0.005
Postoperative length of stay, median (range), day	4 (2–13)	5 (2–147)	<0.001
Conversion to laparotomy, *n* (%)	1 (1.4)	6 (4.0)	0.477
Placement of drainage tube, *n* (%)	8 (11.5)	52 (35.3)	<0.001
Postoperative complications			
Grade 2 or more in C-D classification, *n* (%)	6 (8.6)	39 (26.5)	0.002
Grade 3 in C-D classification *, *n* (%)	1 (0.5)	18 (12.2)	0.009
Intraabdominal abscess, *n* (%)	0	16 (10.8)	0.004
Superficial infection, *n* (%)	0	7 (4.7)	0.065
Paralytic ileus, *n* (%)	2 (1.7)	10 (6.8)	0.242
Readmission, *n* (%)	0	11 (7.4)	0.019

SA, simple appendicitis; CA, complicated appendicitis; LA, laparoscopic appendectomy; C-D, Clavien-Dindo; * SA group included leakage from appendix stump (*n* = 1). CA group included intraabdominal abscess (*n* = 12), superficial infection (*n* = 1), hematoma (*n* = 1), paralytic ileus (*n* = 1), strangulated ileus (*n* = 1), ventral hernia (*n* = 1) and respiratory failure (*n* = 1); Pearson’s chi-square test was used to assess nominal variables and continuous data were compared using the Mann–Whitney U test.

**Table 3:** The results of the univariate and multivariate analyses of prognostic factors associated with developing intraabdominal abscess in the patients with simple and complicated appendicitis.

	No	IAA Positive *n* = 16	IAA Negative *n* = 200	Univariate Analysis *p*	Multivariate Analysis	
					Odds Ratio (95% CI)	*p*
**Gender**				0.283		
Male	135	12	123			
Female	81	4	77			
**Age**				0.304		
<40	121	7	114			
>40	95	9	86			
**ASA-PS classification**				0.983		
1	149	11	138			
2 or 3	67	5	62			
**Onset to surgery**				0.011		0.708
<3 days	154	7	147		1	
>3 days	62	9	53		1.282 (0.349, 4.707)	
**Preoperative WBC**				0.237		
<18,000 /μL	173	11	162			
>18,000 /μL	43	5	38			
**Preoperative CRP**				0.205		
<20 mg/dl	195	13	182			
>20 mg/dl	21	3	18			
Appendicolith *				0.102		
Absent	123	6	117			
Present	93	10	83			
Periappendiceal fluid *				0.003		0.371
Absent	117	3	114		1	
Present	99	13	86		1.960 (0.448, 8.577)	
Abscess *						0.599
Absent	189	9	180	<0.001	1	
Present	27	7	20		1.449 (0.363, 5.780)	
Free air *				<0.001		0.007
Absent	194	8	186		1	
Present	22	8	14		5.427 (1.586, 18.57)	
SBO *				0.093		
Absent	199	13	186			
Present	17	3	14			
**Operation time**				0.023		0.971
<120 min	188	11	177		1	
>120 min	28	5	23		1.027 (0.247, 4.261)	
**Placement of drainage tube**				<0.001		0.081
Absent	156	4	152		1	
Present	60	12	48		3.675 (0.849, 15.92)	

IAA, intraabdominal abscess; CI, confidence interval; ASA-PS, American Society of Anesthesiologists physical status; * judged by preoperative CT image; Pearson’s chi-square test was used for univariate analyses and multivariable logistic regression analysis was performed to determine independent predictors of outcomes.
